# Static Loads Influence on Modal Properties of the Composite Cylindrical Shells with Integrated Sensor Network

**DOI:** 10.3390/s23063327

**Published:** 2023-03-22

**Authors:** Aleksey Mironov, Andrejs Kovalovs, Andris Chate, Aleksejs Safonovs

**Affiliations:** 1 D un D Centrs, LV-1021 Riga, Latvia; aleksei@ddcentrs.lv (A.M.); aleksejs.safonovs@edu.rtu.lv (A.S.); 2 Institute of Materials and Structures, Riga Technical University, LV-1048 Riga, Latvia; and_cate@latnet.lv

**Keywords:** axial tension load, composite structure, operational modal analysis, piezoelectric films, sensors, natural frequencies

## Abstract

This paper presents the results of experimental and numerical studies of the dynamic parameters of composite cylindrical shells loaded under axial tension. Five composite structures were manufactured and loaded up to 4817 N. The static load test was carried out by hanging the load to the lower part of a cylinder. The natural frequencies and mode shapes were measured during testing using a network of 48 piezoelectric sensors that measure the strains of composite shells. The primary modal estimates were calculated with ARTeMIS Modal 7 software using test data. The methods of modal passport, including modal enhancement, were used to improve the accuracy of the primary estimates and reduce the influence of random factors. To estimate the effect of a static load on the modal properties of a composite structure, a numerical calculation and a comparative analysis of experimental and numerical data was carried out. The results of the numerical study confirmed that natural frequency increases with increasing tensile load. The data obtained from experimental results were not fully consistent with the results of numerical analysis, but showed a consistent pattern, repeating for all samples.

## 1. Introduction

In recent years, a large number of research was carried out on isotropic and orthotropic cylindrical shells, which are widely used in modern industry. Owing to their low weight and high strength, they are one of the most common structural members applied in aerospace, mechanical, marine, and civil engineering structures.

However, in the process of operation and the external action of the environment, the structural health changes, which leads to a decrease in reliability. Therefore, in order to ensure reliable, safe, and long-term operation of the structure, it is necessary to monitor its structural health. One of the popular methods for structural health monitoring is based on the vibration approach.

Over the last decades, many books, papers, and scientific reports were published on the vibrational behavior of cylindrical shells, which promoted the development of many theories. A comprehensive generalization and discussion of shell theories, including information on natural frequencies and modes of vibrations, was performed by Liessa [1] in 1973. In this monograph, he selected the theoretical and experimental results of about 1500 scientific papers and reports. A significant contribution to the dynamic theory of shells was also made by Amabili [2], Flugge [3], Donnell [4], Armenàkas [5], Mushtari [6], et al.

It is well-known that the modal parameters of a cylindrical structure are affected not only by its geometry and material, but also by an impact of initial axial forces. The effect of static axial load on the nonlinear vibrations of shells has long been considered an important problem [7]. According to the studies carried out, the natural frequencies and modes of vibrations for a prestressed system differ from those of an unstressed system [8]. Having considered vibrations of the beam, subjected to an influence of a tensile force directed along its axis, the dependence of the natural frequency on the load was revealed [9,10,11,12,13]. With an increasing tensile force, the frequencies of vibrations increase, whereas a compressive force leads to their decrease. If the compressive force reaches a critical value of buckling, the frequencies go to zero.

An investigation of the influence of internal stresses induced by axial load on the dynamic response of plates and shells was considered by Soedel [14]. Using the simplified Donnell-Mushtari-Vlasov theory, an expression for the natural frequency for a cylindrical shell was obtained, depending on the tensile or compressive force. The natural frequency of the cylindrical shell increases with an increasing tensile load and decreases with the compressive one. To date, many studies have been carried out related to the vibrations of cylindrical shells under action of a compressive load, which include experiments, as well as analytical and numerical methods of calculation [15,16,17,18].

However, much less attention has been paid to studies of an influence of tensile force on the natural frequencies and modes of vibrations of a cylindrical shell. A conical shell under action of axial loads was presented in [19]. The results were obtained for conical shells with different geometries and boundary conditions. It was established that axial tension of a cylindrical shell increases natural frequencies, while axial compression reduces them. Bedri performed a modal analysis of a prestressed metal cylindrical cone [20], using the ANSYS finite element program, and showed the dependence of natural frequencies of the model on the axial load, confirming the increasing natural frequency on the longitudinal tensile load. The literature analysis carried out testifies that the correct assessment of the natural frequencies and modes of vibrations of a composite structure requires not only the initial stiffness of the system, but also the additional stiffness caused by the effect of prestressing due to the applied load.

This work is part of a project aimed to develop the prototype system for monitoring serial or mass-produced structures. The basic assumption is that any change in a structure modifies its physical properties and thus, the modal parameters, namely, frequencies and modes of vibrations and damping [21]. Using the relationship between the modal parameters and mechanical properties, a change in the structure health can be estimated from changing its modal parameters [22].

During vibration control of a structure, a large amount of data is generated, so there is a necessity for post-processing by evaluating the experimental model of the structure based on the data obtained. The process of forming a model from test data is called system identification. There are several possible ways to solve the problem of the identification of dynamic parameters, in particular, experimental modal analysis (EMA) and analytical method. As a rule, EMA is performed by determining the response to a known excitation of the system. However, for a number of systems, it is impossible to determine the initial excitation. If only the response is known, the identification of the dynamic parameters of the system is performed by means of operational modal analysis (OMA).

The main techniques used in OMA are also divided into the groups of frequency and time domain methods. In the first case, the representation of the signal in the frequency domain shows the dependence of its physical parameters on frequency. In the time domain, a signal is represented as a dependence of its physical parameters on time [23]. Some of the early applications of the operational modal analysis were the determination of the modal parameters of an aircraft in flight [24], the modal analysis of testing a spacecraft during launch [25], and the modal testing of engines during launch and shutdown [26]. Later, the method was used to study the modal parameters of cars [27]. OMA is one of the most popular methods in the field of civil engineering, which has been applied to bridges, roofs, stone towers, offshore platforms, and high-rise buildings [28,29,30]. Recently, OMA was widely used to determine the modal parameters of wind turbines [31,32].

To estimate the modal structural health using OMA methods, it is necessary to measure its response to external excitation over a large number of degrees of freedom (DOF). This means the structure has to be equipped with a large number of sensors. The use of expensive calibrated accelerometers as sensors makes the system too expensive for mass applications since each of them requires a dedicated measurement channel. Therefore, for mass-produced objects, the cheap and small sensors are needed, which allow for the reduction of the number of measurement channels for monitoring systems. Alternatively, vibration signals can be measured by cheap film sensors. The film sensor placed on a surface generates a signal associated with tensile or bending strains of a vibrated structural element. There are film sensors like wire strain gauges and piezoelectric film sensors. Today, piezoelectric film sensors (piezo films) are mainly applied in tactile interfaces of computer systems or alarm and security systems. Due to negligible mass and dimensions of piezo films, they practically do not affect the modal properties of the object and could be easily integrated. The film sensor may be manufactured as pre-formed flexible skins that adapt to the surface of mass-produced construction. Due to the above benefits, the film sensors can be used instead of accelerometers and contribute to the promotion of vibration monitoring.

This study is part of the project for developing the technologies for structural health monitoring (SHM) of serial structures, using the methods of operational modal analysis. The two main elements of the novelty of this project, implemented in the framework of this study, are the use of piezoelectric films as sensors and the modal passport. This new application of such sensors for SHM is based on their low cost and negligible mass. The modal passport is a set of methods and calculations of diagnostic parameters of working structures. A modal passport enables, using the modal properties common to serial structures, the monitoring of each particular specimen. As external conditions may vary for different specimens, the influence functions are used to take into account the actual conditions. Influence functions are general for identical structures and are being determined by testing the set of such structures, taking into account the external conditions for monitoring the health of the particular specimen. The current work is devoted to the study of the influence of a specific factor—tensile load—on the modal properties of the same type of specimens. Having generalized this influence over all specimens of the series, we obtained an influence function that can be used for SHM within the framework of the modal passport.

## 2. Materials and Methods

### 2.1. Specimens and the Test Bench

A cylindrical composite construction, fitted with an integrated system of piezoelectric film sensors, was used as the object (specimen) for investigating modal properties. Each specimen consisted of a composite cylinder made of fiberglass and epoxy resin; top and bottom flanges made of laminated plywood; networks of piezoelectric strain sensors with connecting wires and D-SUB connectors.

#### 2.1.1. Specimens

**Figure 1:**
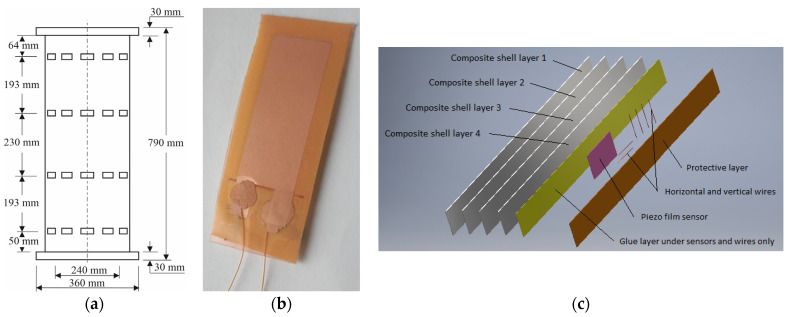
The specimen: (**a**) design of cylindrical shell and location of the piezoelectric sensors; (**b**) piezoelectric sensor; (**c**) exploded view for cylinder wall element.

The overall dimensions of the specimen and its design are presented in Figure 1. Technologically, a serial sample was formed from a composite cylindrical shell with a network of piezoelectric sensors glued to it, covered with a protective composite layer and two ring-shaped flanges.

A circular cylindrical shell with a 300 mm diameter and 760 mm height was made of eight layers of unidirectional fiberglass (Figure 1a), with a density of 300 g/m^2^ with ±45° fiber orientation and LG 385 epoxy resin with HG 385 hardener. The network of 48 piezoelectric sensors (Figure 1b) and wires was placed on the surface of the composite cylinder and measured the strains of the shell. The sensors were located around the circumference in four annular sections of the cylindrical shell, with 12 in each.

To protect the sensor network, a protective layer of satin weave glass fiber, with a density of 50 g/m^2^, was glued using the same epoxy resin and hardener (Figure 1c). The total wall thickness of the composite shell, after its manufacture, was 1.45 ± 0.05 mm. At the last stage of serial sample production, to ensure its mechanical strength, top and bottom edges of the sample were glued into the grooves in the plywood flanges (30 mm thickness). The depth of the grooves was 15 mm. Active height of the circular cylindrical shell between plywood flanges was 730 mm. The top and bottom flanges had the same construction with 6 holes (6 mm diameter), with centers evenly distributed circumferentially. The upper flange was intended for fastening to the attachment point of the test stand; the lower one was for hanging a basket with a static load. The total mass of the composite cylinder, with sensors and wires and two plywood flanges, was 4.58 ± 0.09 kg.

Each specimen had its own integrated sensor network, which outputs a signal through four D-SUBs, which came to a measuring system for signal recording and storage. The sensor network of each sample was formed from 48 sensors connecting them to the terminals of the conductors: four terminals and wire harnesses with connectors. The conductors were glued to the sensors with a special conductive adhesive, after which the sensor was covered with a non-conductive insulating tape. Sensors were installed on the cylindrical part of the sample in accordance with the pre-made markup, corresponding to the diagram in Figure 1a.

#### 2.1.2. The Test Bench

**Figure 2:**
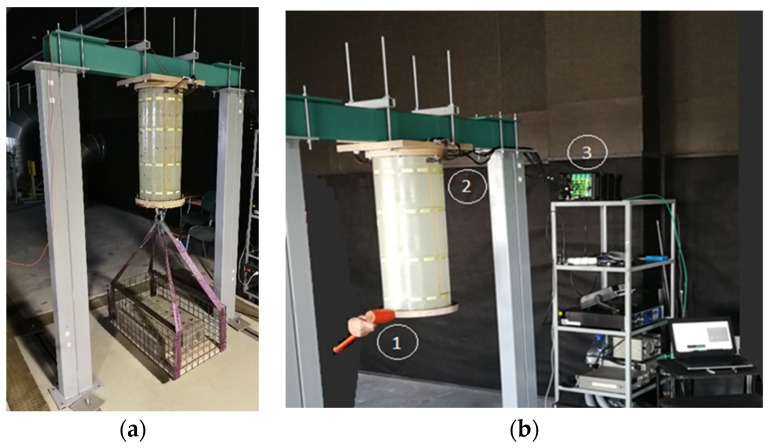
Test setup: (**a**) specimen with suspended weight; (**b**) plastic hammer for actuation.

For static load testing, the special test bench was used and fixed on a vibration-isolated base (Figure 2a). The specimen was placed vertically on the test bench and fixed through the top flange. The static load test was carried out by hanging the load to the lower flange, through an additional element, with an eye fixed on it for attaching slings.

The load required for the test was collected using the appropriate number of weights, each 33.7 ± 2.5 kg. Since the upper load limit was 5000 N, the maximum load of the 14 specimens was 4817 N. The state of the specimen loaded by an empty basket was considered as the reference one.

#### 2.1.3. Testing and Measurements

To simulate random excitation of the specimen, a plastic inertial hammer (1) was used (Figure 2b). Impacts were made in three directions, moving around the lower flange. Upon impacts on the lower flange, the specimen’s natural vibrations were excited. Signals through four D-SUBs of the specimen and connecting cables (2) came to the measurement system (3).

The latter included the 48 channel measurement unit and the laptop with data recording software. The data collected during the test was saved as the file, with identification name. Three tests were used for each load grade.

### 2.2. Modal Data Development

To assess the influence of static loading on modal properties of the specimen, high accuracy of modal estimation is required. Within the preliminary study [33], when the prototype of typical specimens was tested, the standard software ARTeMIS Modal 7 for OMA (Artemis) was applied for modal data development. As input data, this software, using digitalized signals of sensors, provides modal identification and estimates modal parameters (frequency, damping, and shape) for each identified mode. As the preliminary study showed, primary estimates of the standard software from single test data were rough and had essential spread between similar estimates. To increase the accuracy of modal parameters estimation, the techniques of modal passport (MP) were applied. The MP techniques consider the primary estimates of Artemis as input data and ensure the accuracy of computed modal parameters, reducing its uncertainty.

The MP approach allows application of modal parameters for the structural monitoring of serial structures. The MP considers both the common modal properties of serial samples and specialties of the particular one. The MP provides solutions for two principal tasks: computation of accurate modal parameters and its application for monitoring. For computing modal parameters, the MP applies the modal enhancement procedures to estimates of primary modal that typical OMA software identifies [34]. For monitoring the serial sample, the MP contains typical and individual parts.

#### 2.2.1. Typical MP

The typical part of MP includes the modal model of the typical design, the testing procedure, the set of modal parameters, and the influence functions, common to all samples of the typical design. For monitoring tasks, the MP uses common thresholds of a reference state. The modal model has *N* degrees of freedom (DOFs) matched to *N* sensors placed on each serial sample. The signal data recorded the procedure, taking into account the specialty of ambient and operational excitations that are typical for serial samples. For the test case considered in this study, the typical testing procedure complemented the recording procedure to simulate ambient excitation. The set of modal parameters is common for samples of typical design. The influence functions allow the consideration of an external influence; in the present work, the static load providing tensile stresses was the factor of influence. The thresholds of diagnostic parameters characterize the boundaries of reference condition for any series sample.

#### 2.2.2. Individual MP

The individual MP applies the procedures and the data (typical modes and thresholds) of the typical MP to the particular sample (specimen in this study). The specimen is equipped with a measurement system matching the typical modal model, and it is tested in accordance with the typical measurement and test procedure. In this study, the test procedure described conditions of hammer excitation and signal recordings. For computing the primary modal estimates, the typical OMA algorithms were applied to recorded data. Then, modal enhancement procedures were used for modal uncertainty reduction. The enhanced modal parameters, in this study, were used for influencing function evaluation.

#### 2.2.3. Primary Modal Estimates

The software of the Artemis package calculates the primary estimates of modal frequencies, damping, and eigenvectors, using five modal estimation techniques (estimators). Therefore, the primary modal estimates of a single test of a specimen includes five groups of modal parameters. A random part of vibration signals causes the high uncertainty of primary modal estimates. The random component depends on manual actuation of a specimen and ambient factors, like acoustic noises and vibrations. Additionally, mismatched modal estimates, provided by different OMA estimators, cause uncertainty. There are also measurement and calculation errors that increase the uncertainty of primary estimates. The modal enhancement allows for a reduction of the uncertainty of modal parameters estimated from measured data.

#### 2.2.4. Modal Enhancement

Modal enhancement is similar to the enhancement of the vibration waveform in vibration diagnostics [35], but is performed in an imaginary *N*-dimensional space, rather than in phase domain. The modal enhancement requires the sequence of modal estimate transformations, including normalizing the modal shape (eigenvectors), modes grouping, and the phase alignment of eigenvectors.

Unequal scales of eigenvectors, provided by different estimators, require normalizing the modal shape for further development. The basis of normalization is the vector sum of the eigenvectors. The normalization brings all eigenvectors to a common (−1.0… 1.0) range.

The mode grouping procedure provides for the similar mode identification, the stable mode selection from the totality, and their grouping. To check the similarity of a pair of modes, the Modal Assurance Criterion (MAC) [36] was calculated for their eigenvectors. MAC values close to unity testify about the similarity of the mode shapes, whereas zero indicates its absence. In the current study, the modes were considered similar ones if the MAC value was greater than 0.98. If three or more modes were similar, then their mode was considered as stable, and they were combined in a group of common modes. As a result of modal grouping, *K* unique mode groups were formed, each of which included $M_{k}$ similar modes.

Once the eigenvector phases in the group were matched, the modal enhancement could be performed. Since different applied estimators can reflect the same mode shape in the opposite phases, phase matching was required. If the check of phase coincidence for any eigenvector in the group turns out to be negative, then the values of such eigenvector are inverted.

The enhancement of normalized, grouped, and matched modal parameters provides higher accuracy for assessments of modal parameter over $M_{k}$ modes of *k*th group. The dependence of three parameters of each *m*th mode on static load were studied: frequency ${\bar{f}}_{m}^{e}$, damping ${\bar{d}}_{m}^{e}$, and shape intensity $si_{m}^{e}$. The shape intensity parameter is a derivative from the modal shape eigenvector $s_{n}^{m}$, consisting of *N* elements. Instead of an eigenvector to simplify characterizing the modal shape, inherent to the considered thin-walled cylinder, the scalar parameter was applied:

(1) $$si_{m}^{e}=\frac{1}{N}\left(\sum_{n_{+}}^{N_{+}}s_{n}^{m}-\sum_{n_{-}}^{N_{-}}s_{n}^{m}\right),$$

where $n_{+}$, $n_{-}$ are the numbers of DOFs with positive and negative means, $N_{+}$, $N_{-}$ are the quantity of positive and negative means of eigenvector’s elements of *m*th mode.

The enhanced mean of modal parameters was calculated as arithmetic mean of $M_{k}$ estimates of *k*th group for *m*th mode:

$${\bar{f}}_{m}^{e}=\frac{1}{M_{k}}\overset{M_{k}}{\underset{1}{\sum }}f_{m}^{k},$$

(2) $${\bar{d}}_{m}^{e}=\frac{1}{M_{k}}\sum_{k=1}^{M_{k}}d_{m}^{k},$$

$$si_{m}^{e}=\frac{1}{M_{k}}\sum_{k=1}^{M_{k}}si_{m}^{k}.$$

The enhanced modal parameters, computed from the test data using Equation (2), reflect the modal properties of the tested specimen with the fixed load state. When the modal parameters of the specimen are computed for complete range of static loads, the appropriate influence function for this specimen can be estimated. Preliminary analysis of the modal data had indicated the modal frequency dependence on static load as closed to linear. This allows linear approximation of the dependence between the static loads and the modal parameters.

Influence functions of modal parameters, for instance, frequency on temperature, may be described using analytical expressions for *m*th mode:

(3) $$f_{m}\left(T\right)=F_{m}^{T}\left(T\right)+f_{m}^{o}.$$

In this equation, the temperature influence function $F_{m}^{T}$ is common for all specimens (serial samples) in the determined range $T_{low}\ldots T_{high}$. Since the modal frequencies may vary between specimens, the frequency $f_{m}^{o}$ is specific for the particular specimen in the reference state. If the dependence of the modal parameter on the static load is close to linear, like $f_{m}\left(T\right)=K_{m}^{T}\left(T\right)+f_{m}^{o}$, the least squares method is used to find the regression coefficient $K_{m}^{f}$. In case of zero initial load, $L_{0}=0$ and *N* means measured frequency $f_{m}^{i}$ and load $L_{i}$:

(4) $$K_{m}^{f}=\left(N\sum_{i=1}^{N}L_{i}f_{m}^{i}-\sum_{i=1}^{N}L_{i}\times \sum_{i=1}^{N}f_{m}^{i}\right)/\left[N\sum_{i=1}^{N}L_{i}{}^{2}-{\left(\sum_{i=1}^{N}L_{i}\right)}^{2}\right].$$

The regression coefficient of the typical influence function $Ktyp_{m}^{f}$ may be determined, experimentally, by testing a series of test samples (five in this study). Since these coefficients vary between specimens, the typical (common) linear regression coefficient for *m*th mode can be found as average:

(5) $$Ktyp_{m}^{f}=\sum_{Sp=1}^{M_{Sp}}K_{mSp}^{s}.$$

The influence function determined for the typical design, as mentioned above, can be used for monitoring any specimen of this design. The value of the modal parameter of an operating structure is evaluated under arbitrary conditions, for instance load *L*. In monitoring tasks, the modal parameter is compared with the threshold set for reference conditions ($L_{0}$). Therefore, monitoring requires an estimate of the modal parameter corresponding to the reference state. For example, the frequency value $f_{m}^{k}{}_{0}$ of the *m*th mode of the *k*th sample at the reference state $\left(L_{0}\right)$ can be estimated from the frequency $f_{m}^{k}$, estimated at arbitrary load using a typical regression coefficient:

(6) $$f_{m}^{k}{}_{0}=f_{m}^{k}-Ktyp_{m}^{f}\left(L-L_{0}\right).$$

### 2.3. Numerical Modelling of Specimens

To estimate the effect of a static load on the modal properties of a composite structure, a numerical calculation and a comparative analysis of experimental and numerical data was carried out.

#### 2.3.1. Prestressed Modal Analysis

Prestressed modal analysis calculates the natural frequencies and mode of vibrations of the model, using the static analysis results, and consists of two stages. Firstly, the static problem of loading the structure is solved, and then a modal analysis is performed using the resulting stress fields to determine the natural frequencies of the vibrations.

To solve the modal analysis problem, it is assumed that the external forces and damping are equal to zero. The equation of free vibrations of the structure in matrix form has the form:

(7) $$\left[K\right]\left\{u\right\}+\left[M\right]\left\{\ddot{u}\right\}=\left\{0\right\},$$

where $\left\{u\right\}$ is the vector of displacements of the whole body, $\left\{\ddot{u}\right\}$ is the vector of accelerations of the body points, [*K*] and [*M*] are the matrices of stiffness and mass of the whole body.

For a linear system, free vibrations are harmonic and can be written as:

(8) $$\left\{u\right\}={\left\{\varphi \right\}}_{i}\cos\omega_{i}t,$$

where ${\left\{\varphi \right\}}_{i}$ is the first eigenvector representing the shape (mode) of vibrations *i*th natural frequency, $\omega_{i}$ is the *i*th natural frequency, *t* is the time.

Substituting Equation (7) in Equation (8), we obtain:

(9) $$\left(\left[K\right]-\omega_{i}^{2}\left[M\right]\right){\left\{\varphi \right\}}_{i}=\left\{0\right\}.$$

This equality is satisfied if ${\left\{\varphi \right\}}_{i}$ = {0} or if the determinant of the matrix $\left(\left[K\right]-\omega_{i}^{2}\left[M\right]\right)$ is equal to zero.

Under modal analysis of a prestressed structure, time-constant stresses are taken into account, which can affect the natural frequency of the structure. Firstly, it is necessary to analyze the static structure, calculated as follows:

(10) $$\left[K\right]\left\{x\right\}=\left\{F\right\},$$

where $\left\{F\right\}$ is the force vector, $\left\{x\right\}$ is the displacement vector.

Nonlinear static analysis is performed to determine the change in geometry and the stresses arising in the model. Then, the additional stiffness matrix $\left[S\right]$ is calculated from the stresses, and the final equation for calculating natural frequencies and modes of vibrations of the prestressed structure takes the following form:

(11) $$\left(\left[Kn+S\right]-\omega_{i}^{2}\left[M\right]\right){\left\{\varphi \right\}}_{i}=\left\{0\right\},$$

where $\left[Kn\right]$ is the stiffness matrix, taking to account the change in geometry, $\left[S\right]$ is the so-called geometric stiffness matrix, obtained on the basis of the prestress tensor and the nonlinear part of the strain tensor [37].

#### 2.3.2. Finite Element Model of a Composite Cylindrical Shell

**Figure 3:**
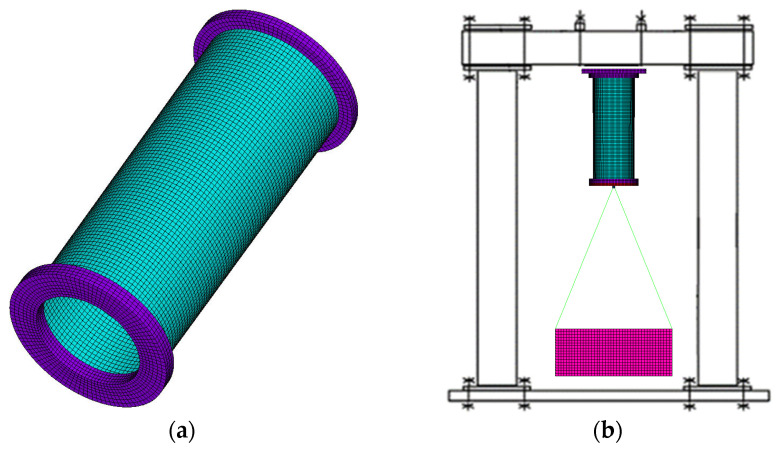
(**a**) Finite element model of manufactured composite cylinder; (**b**) conditions of full-scale experiment.

A 3D finite element (FE) model of a composite cylinder was created using the commercial finite element software ANSYS 16.0 (Figure 3a). When modelling the problem in 3D formulation, linear 4-node SHELL181 elements were used to model a multi-layered composite cylindrical shell and linear 8-node SOLID185 elements were used to model the rigid plywood rings, the top and bottom plywood cover plates, and suspended basket with a load. The connection with the basket and composite cylinder was modelled by BEAM188 element.

The top plywood cover was used to attach to the top crossbeam of the test stand. The calculation was performed with a cantilever restraint of the structure, simulating the conditions of full-scale experiments (Figure 3b).

Before numerical calculations, the mesh convergence analysis was carried out to obtain results with acceptable accuracy. The mesh convergence study was carried out for the element sizes of 5 × 5, 10 × 10, 15 × 15, and 20 × 20 mm on a composite cylinder shell, without rigid plywood flanges [38]. Free–free boundary conditions were used for the calculation of the first 22 natural frequencies in the composite cylinder. The mode shapes at the element size of 5 × 5 and 10 × 10 mm were similar, which testifies the good correlation of the results obtained. During calculation, mode shapes changed, beginning with the element size of 15 × 15 mm and 20 × 20 mm, after 9th and 3rd mode shapes, respectively. Thus, the finite element model, with element size 10 × 10 mm, was chosen for ensuring the high accuracy of future research and decreasing the time of calculation.

#### 2.3.3. Calibration of Numerical Model

Taking into account the complexity of the composite structure and the technology of its manufacture, it was necessary to preliminarily calibrate the finite element model of a composite cylindrical shell with flanges. The procedure used to calibrate the model was based on the parametric model of the composite structure and the minimization of the function on the discrepancy between the experimental and calculated natural frequencies [39], as follows:

(12) $$\Phi_{i}\left(x\right)=\sum_{i=1}^{m}\frac{{\left(f_{i}{}^{exp}-f_{i}{}^{FEM}\right)}^{2}}{{\left(f_{i}{}^{exp}\right)}^{2}}\Rightarrow min,$$

where $f_{i}{}^{exp}$ and $f_{i}{}^{FEM}$ are the frequencies of vibrations obtained experimentally and numerically, *i* is the number of natural frequencies used in this method.

**Figure 4:**
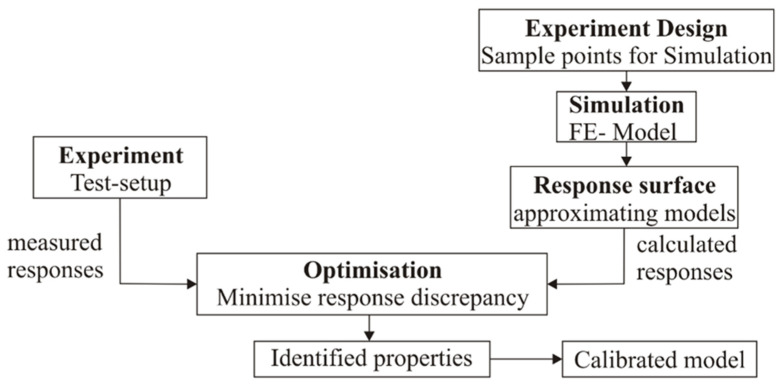
Calibration method of the numerical model [41].

The numerical-experimental method for determining the properties of a composite material and calibrating a finite element model consists of several stages (Figure 4). At the first stage, the frequencies and modes of natural vibrations of the composite structure, without the bottom plywood cover for the load fastening and upper plate, are experimentally determined. At the second stage, the estimated values of the elastic constants of the composite shell are selected and an experimental plan is created. To develop an experimental plan and determine the properties of the material of a multi-layered composite shell, planning was used with a complete enumeration of possible options for identification parameters, i.e., a Latin cube (LH). According to the LH plan, the experiment points are distributed as evenly as possible.

The properties of birch plywood are assumed to be fixed: longitudinal modulus of elasticity *E* = 17 GPa, Poisson’s ratio *ν* = 0.35, and density ρ = 810 kg/m^3^. The mechanical properties of spruce wood are taken from EN 338 [40] timber grade standard. Plywood layers are neglected in this research.

The accepted value for the density model is overestimated compared to the density of the plywood material (ρ = 685 kg/m^3^), since the cylinder’s edges are fixed in the plywood flange, and its density increases due to the use of a mixture of glue and sand in the gluing zone.

Numerical values of natural frequencies, obtained by the finite element method at the design points of the experimental plan, were used to construct approximating functions. The identification results obtained for the composite shell are presented in Table 1.

The obtained properties of the composite material were checked by comparing the natural frequencies and modes of vibrations of the composite structure, calculated in the ANSYS finite element program $f_{i}^{FEM}$, and experimentally determined $f_{i}^{EXP}$ by the following expression:

(13) $$\Delta_{f}=\frac{\left|f_{i}^{FEM}-f_{i}^{EXP}\right|}{f_{i}^{EXP}}\times 100.$$

The modes and corresponding natural frequencies, determined numerically, were compared with experimental ones for Specimen 5 with clamped–free boundary conditions in Table 2. Mode shapes for the numerical and experimental models were the same. The greatest percentage difference in frequencies was observed for only two mode shapes: (1;1) and (1;2).

**Figure 5:**
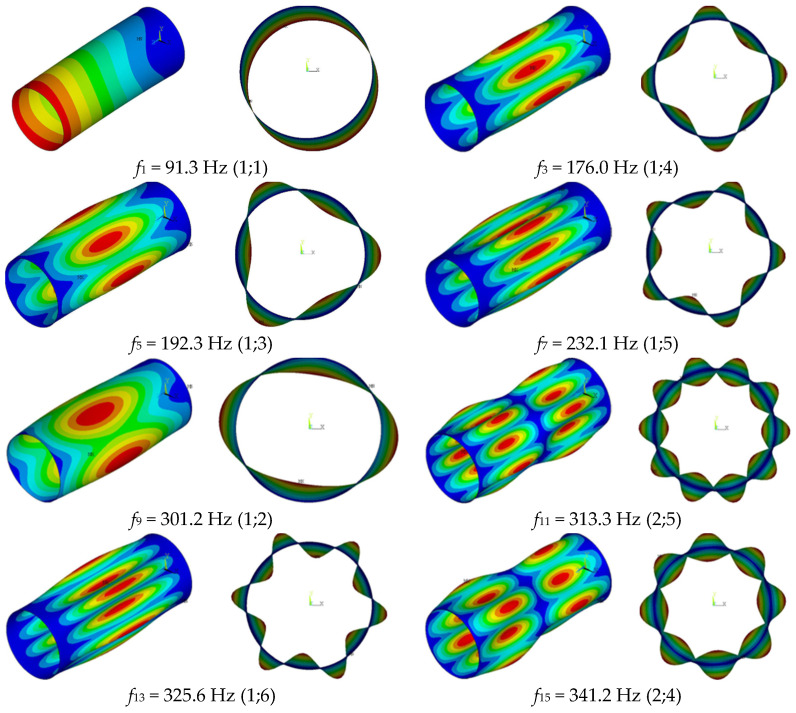
Fundamental vibration mode shapes and corresponding frequencies.

Fundamental modes of the vibrations of the cantilever composite cylindrical shells, without plywood, are shown in Figure 5. It can be seen that the shell has multiple natural frequencies, which is typical for structures with axial symmetry. The modes of vibrations corresponding to multiple frequencies were shifted by a quarter wavelength along the circumferential coordinate, relative to each other. Therefore, Figure 5 shows the modes of vibrations for the first multiple of the frequency.

The modal frequency, damping, and mode of vibrations varied between specimens, due to structural deviations that were caused by hand-made manufacture. More detailed data about the variation of modal parameters between specimens were presented in [34].

## 3. Results

The experimental study was devoted to analyzing the effect of static loading on the modal properties of series specimens. The objective was to practically verify the proposed method of estimating the dependence of modal parameters on load, which is common for all specimens of a given type. The solution of the stated task included the stages of modelling and experimental investigation, applying OMA and MP methods. Modelling made it possible to identify the modes of the specimen’s model in a given frequency range and to numerically estimate the influence of static loading on the modal frequencies. Experimental investigation was carried out for five specimens, for which the dependences were estimated for modal frequency, damping, and the shape of sustainable modes of each specimen.

### 3.1. Modal Data Development

Before the experimental test of composite structures, the effect of static loads was studied numerically by means of the ANSYS finite element program. The procedure for performing a modal analysis of a prestressed composite structure in the ANSYS finite element program is concerned with the influence of the initial stress from the tensile force on the dynamic response of the structure. Firstly, a nonlinear static calculation of the model by implicit solver was carried out, using the option that takes into account the effect of the resulting stresses from the axial load. Static analysis was done using an implicit solver in ANSYS. Then, the natural frequencies and mode of vibrations, taking into account the prestressed state, were determined by modal analysis, accounting for the results of the static analysis.

The calculation of the composite specimen was performed under the condition of the rigid fixation of the top plywood end. Load application was modelled by means of cover plate connection with the basket. The acceleration of gravity 9.81 m/s^2^ was applied to simulate the effect of axial tensile force on the composite specimen, which corresponded to the test conditions for a serial specimen. The tensile force, in numerical calculations, is changed by changing the density of the hanging basket.

A nonlinear static calculation shows that there are radial forces from flanges that are caused by static load. These forces compress ends of the cylinder part, leading to compressive strains in the circumferential direction. There is also an uneven distribution of strains along the height of the cylinder part. So, in the cylinder shell, tensile stresses in the axial direction are combined with compressive stresses in the circumferential direction.

The calculation results of natural frequencies are shown in Table 3 for the first multiple of the frequency. It can be seen that load increase leads to an increase of natural frequencies. The exception is the first bending frequency, which decreases with an increasing load.

**Figure 6:**
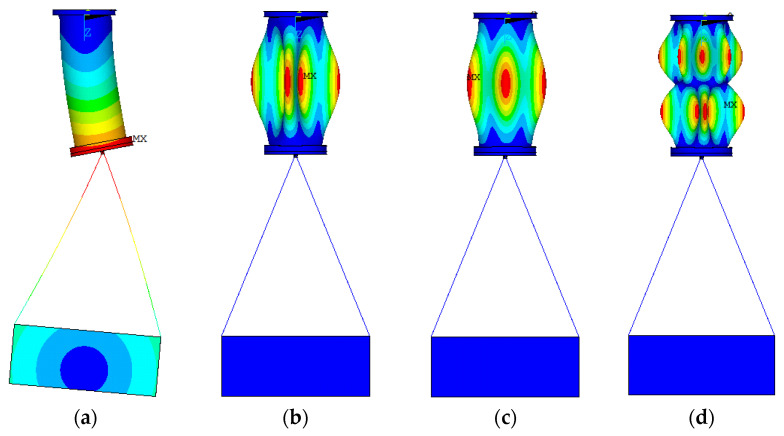
Fundamental vibration mode shapes: (**a**) mode (1;1); (**b**) mode (1;4); (**c**) mode (1;3); (**d**) mode (2;5).

Examples of the fundamental modes of vibrations of the cantilever composite specimen, with hanging basket, are shown in Figure 6. Figure 6a illustrates that the bending mode shape occurs in the cantilevered specimen, in combination with the basket, that can lead to a decrease of frequencies. For other mode shapes, the vibration only occurs in the cantilevered composite specimen, without interaction of the basket.

The modal frequency percentage change is calculated using a ratio of corresponding modal frequencies for prestressed composite construction, with a reference tensile force of 167 N, and with increased tensile force:

(14) $$\Delta_{i}=\left|\frac{f_{i}-f_{167}}{f_{167}}\right|\cdot 100\%,$$

where $f_{167}$ is the modal frequency of prestressed composite structure, subjected to the tensile force of 167 N, and $f_{i}$ is the modal frequency of prestressed composite structure, subjected to increased tensile force.

**Figure 7:**
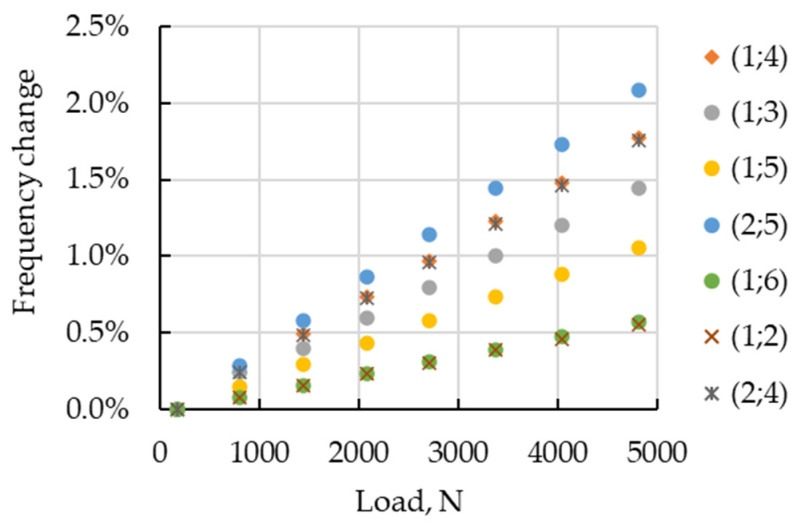
Percentage change increase versus tensile force.

The percentage effect of the tensile force on the frequencies of vibrations of the composite cylinder, as a function of the applied axial load for each mode of vibrations, is shown in Figure 7. The results, presented in Figure 7, testify that the tensile load leads to an increase in frequencies. The changes are linear. The effect of loading on frequencies is negligible.

### 3.2. Modal Testing Specimens

Modal tests for each of the five specimens were performed according to the methodology presented in Section 2.1. The data files with signals of the sensors were processed, applying OMA and MP techniques, according to Section 2.2. As the result of modal data development, the modal parameters (frequency, damping, and mode of vibration) were obtained for each of the five samples for each tested state.

#### 3.2.1. Model Verification

The effectiveness of the FE model was evaluated by comparing the FEM results with experimental ones for model testing of Specimen 5 in the reference state 167 N (Table 4). The modelling error, with respect to experimentally obtained data, is given in column 6, allowing for the estimation of the effectiveness of the FE model, which also depends on the order of vibration modes.

Comparison showed that the differences of calculated and measured frequencies for the first four paired modes do not exceed 6%. For paired modes (1;6) and (2;4), the percentage differences are less than 4%. However, beginning with the 9th order mode, shapes (2;5) and (1;2) are swapped in the numerical model. Additionally, this order shows the biggest frequency difference between numerical and experimental data: −17%.

#### 3.2.2. Static Load Influence on Frequencies

**Figure 8:**
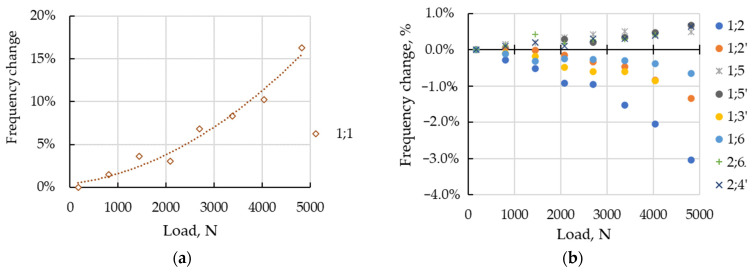
Frequency change under static load for Specimen 1: (**a**) bending mode (1;1); (**b**) shell modes.

The modal parameters’ dependence on static load was only studied for sustainable modes of vibrations. The modes identified in at least half of the tests, with loading grades, were considered sustainable ones. The set of sustainable modes varied for specimens and typically included 6–13 modes. Due to wide frequency range, the frequency change from the reference state was analyzed. The dependence of frequency change to static load, for sustainable modes of Specimen 1, are illustrated in Figure 8.

In contrast to simulated modes, the experimentally estimated frequencies changed differently with loading. The frequency of bending mode (1;1) increased, most notably, up to 16% (Figure 8a). The frequencies of shell modes (Figure 8b) changed differently (−3%…+1%).

**Figure 9:**
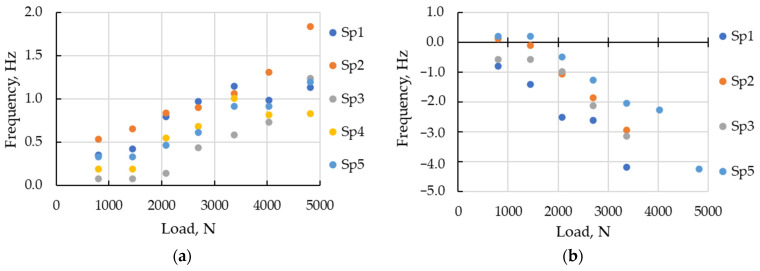
Modal frequency dependence on static load: (**a**) mode (1;5); (**b**) mode (1;2).

Although frequencies of the same mode varied between specimens, its change under load was comparable. Figure 9 illustrates examples of frequency change (different specimens) for two types of modes: with growing frequency (1;5) and decreasing frequency (1;2′).

As an example of typical influence function definition, the frequency dependencies of mode (1;5) is considered in Figure 9a. It is evident that the frequency dependence on load for similar modes of different specimens has close tendencies but has shifted along the frequency axis. Such shift corresponds to a difference of modal frequencies between specimens [34]. These frequency trends were used for defining the influence function considered in Section 2.2. The trends of frequency dependencies for all specimens presented in Figure 9a were close to linear. The regression coefficients of frequency change, calculated according to Equation (4), varied in the range 0.21…0.33$\times 10^{3}{\;\mathrm{HzN}}^{-1}$. The typical linear regression coefficient $Ktyp_{m1-5}^{f}$, computed using Equation (5) as $0.25\times 10^{-3}{\;\mathrm{HzN}}^{-1}$, may be used as the load influence function $f_{m}\left(L\right)$ for typical mode (1;5) for a series of specimens:

(15) $$f_{m}\left(L\right)=F_{1-5}^{L}\left(L\right)+f_{1-5}^{o}=0.25\times 10^{-3}L+f_{1-5}^{o}.$$

As Equation (15) describes the frequency of mode (1;5) for any typical specimen, adding the modal frequency $f_{1-5}^{o}$ of the specimen in reference state to increment $0.25\times 10^{-3}L$, caused by a load change, can be calculated. For a monitoring task, the MP considers the reverse problem: using the measured modal parameter of the structure and predetermined influence function to estimate the modal parameter of the reference state.

Modal frequency estimation, applying the influence functions, includes the error, which corresponds to the spread of the considered trends (between specimens). The error caused by an influence function was calculated as the difference between the experimental frequency and the estimated one with influence function. Since each frequency estimation (by influence function $f_{m}\left(L\right)$) caused an error, the standard deviation of errors (STE) for all loading grade and all specimens was used for error characterization. For the load-frequency of the influence function of modes (1;5) and (1;2′), the STE was estimated about 0.1 and 0.3%, respectively.

#### 3.2.3. Static Load Influence on Modal Damping

**Figure 10:**
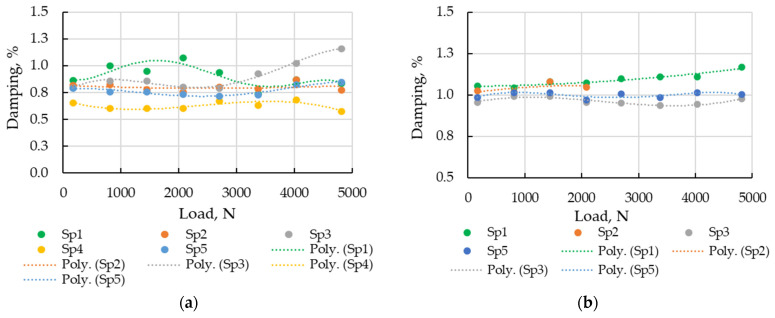
Damping elevation to static load for different specimens: (**a**) mode (1;5); (**b**) mode (1;2′).

The results of processing the experimental data by MP techniques allow for the estimation of the influence of static loads, not only on frequencies of identified modes, but also on their damping and shape. The effect of static loading on damping is more complex and is illustrated in Figure 10 for modes (1;5) and (1;2′).

The dependence of shape intensity on static load is close to linear, so a similar approach to frequency can be used for this parameter. For the load-shape (intensity) influence function of modes (1;5) and (1;2′), the STE was estimated about 0.3 and 0.6%, respectively.

## 4. Discussion

The evaluation of the model effectiveness is illustrated by the data in Table 4. Analysis of the simulated changes of the modal frequencies (Figure 7) and comparison with the analytical conclusions in the Introduction substantiate the increase of frequency with growing the tensile load. The nature of the change of natural frequencies under the influence of the load for the experimentally identified modes (Figure 8) differed from the simulation results (Figure 7). Firstly, the frequencies could not only increase with loading, but also decrease. The greatest differences were observed for modes of the lowest order, in particular, the bending mode, whose frequency increased by 16%. At the same time, the frequency scale of the higher order modes was consistent with the simulation data.

According to the studies, the natural frequencies and vibration modes of a prestressed system differed from those for an unstressed system. However, the experimental values of natural frequencies for some vibration modes, with an increasing applied tensile load, do not agree with the theoretical and calculated values. The increase and decrease of natural frequencies, depending on the mode shape with the tensile load growth, suggests that, under tensile loads, both compressive and tensile stresses simultaneously arise in the cylindrical shell. It is possible to put forward a number of assumptions about the reasons for the discrepancy between the experimental and analytical and numerical results obtained. Due to the imperfection of the hand-layup method of manufacturing cylindrical specimens, its uneven wall thickness can cause a change in the stress-strain state of the cylinder under the action of an axial tensile load.

Natural vibration modes depend on the configuration of a structure and the longitudinal and transverse wave propagation in its material. The tensile stresses of the structure lead to a growth in the speed of wave propagation and increase natural frequencies, whereas compress stresses may have the opposite effect. Depending on the mode shape, the dual influence of positive and negative values of stresses may have different effects. The specimens considered in this study were manufactured manually, and the wall thickness was irregular. This effect led to an increase in the uneven distribution of tensile and compressive stresses in axial and circumferential directions.

A comparative analysis of the modal parameters’ dependence on the static load revealed the trends, which, for each mode type, were common for all specimens. This allowed for the determination of the common (typical) dependence of each modal parameter on static load, which the MP uses as influence functions. In this work, the linear regression coefficients of modal parameters (frequency, damping, and shape intensity) were calculated as the influence functions. These influence functions allow for the recalculation of the value of the modal parameter, determined at one load for another one, including the reference state. Such recalculation is necessary for monitoring in order to compare the estimated modal parameter with the threshold that is set for reference conditions.

It is noted that the assumption of the linearity of the parameter dependence on load was not observed everywhere, especially for damping. In addition, there was a scatter between the dependencies of the same type of modal parameters for different specimens, due to the mass size differences. The error caused by the influence function application allowed for the comparison of the diagnostic efficiency of different modal parameters. The smallest errors characterized using STE were found for parameters of frequency (0.1…0.3%) and of shape intensity (0.3…0.6%). In an order higher (4…16%), the error levels were observed for the damping parameter estimates.

## 5. Conclusions

This paper presents the results of experimental and numerical studies of the dynamic parameters of composite cylinders under a tensile load. The results shown in this study are part of a research project which aims to develop a prototype system for monitoring serial or mass-produced structures, using its modal parameters. The basic assumption is that any change in a structure modifies its physical properties and thus, the modal parameters, namely, frequency, shape, and damping. One of the monitoring problems is an influence of static load on modal parameters that may cause false alarm or target miss.

The following conclusions can be drawn from the results obtained:

1. The preliminary tensile load leads to an increase of the frequency in the numerical model, whose change follows a linear relationship. The differences between the natural frequencies of the structure in the range of applied loads does not exceed 2%.
2. The tensile load influence functions for modal frequency and shape parameters, obtained from the experimental data, were not fully consistent with the numerical analysis, but showed a consistent pattern, repeating for all samples.
3. Commonality of influence functions for single-type structures confirm the possibility of experimentally determining above functions of modal parameters in order to use them for monitoring tasks.

## Figures and Tables

**Table 1:** The identified engineering constants.

Engineering Constants	*E*_1_, GPa	*E*_2_ = *E*_3_, GPa	*G*_12_ = *G*_13_, GPa	*G*_23_, GPa	*ν*_12_ = *ν*_13_	*ν*_23_
Value	40.90	11.14	1.70	3.01	0.416	0.23

**Table 2:** Comparison of the modes of vibrations and corresponding natural frequencies determined numerically and experimentally.

Order	Mode (m;n)	Frequency (Hz)		*Δ_f_* (%)
		Experiment	Simulation	
1	2	3	4	5
1	(1;1)	78.4	91.3	12.9
2	(1;1′)	104.8	91.3	12.9
3	(1;4)	174.5	176.0	0.9
4	(1;4′)	175.9	176.0	0.1
5	(1;3)	185.0	192.3	3.9
6	(1;3′)	185.0	192.3	3.9
7	(1;5)	230.2	232.1	0.8
8	(1;5′)	232.4	232.1	0.1
9	(1;2)	261.9	301.2	15.0
10	(1;2′)	266.2	301.2	13.2
11	(2;5)	315.6	313.3	0.7
12	(2;5′)	319.2	313.3	1.8
13	(1;6)	321.0	325.6	1.4
14	(1;6′)	-	325.6	-
15	(2;4′)	344.5	341.2	1.0
16	(2;4′)	-	341.2	-

**Table 3:** The first numerical modes against the axial static load’s levels.

Order	Mode (*m*;*n*)	Tensile Force, N							
		167	804	1442	2080	2698	3375	4032	4817
		Frequency, Hz							
1	(1;1)	54.1	43.0	34.5	31.1	28.3	26.0	24.2	22.5
2	(1;4)	177.4	177.8	178.2	178.7	179.1	179.5	180.0	180.5
3	(1;3)	200.6	201.1	201.4	201.8	202.2	202.6	203.0	203.5
4	(1;5)	232.4	232.7	233.1	233.4	233.7	234.1	234.4	234.9
5	(2;5)	314.6	315.5	316.4	317.3	318.2	319.1	320.0	321.1
6	(1;6)	325.8	326.0	326.3	326.5	326.8	327.1	327.3	327.6
7	(1;2)	337.8	338.1	338.4	338.6	338.9	339.1	339.4	339.7
8	(2;4)	346.0	346.8	347.7	348.5	349.3	350.2	351.0	352.1

**Table 4:** The first numerical modes against the axial static load levels for Specimen 5.

Order	Experiment		Simulation		*Δ*_*i*_ (%)
	Mode (*m*;*n*)	Frequency (Hz)	Mode (*m*;*n*)	Frequency (Hz)	
1	2	3	4	5	6
1	(1;1)	-	(1;1)	54.1	-
2	(1;1′)	-	(1;1′)	56.9	-
3	(1;4)	179.2	(1;4)	177.4	1.0
4	(1;4′)	182.5	(1;4′)	177.4	2.8
5	(1;3)	189.2	(1;3)	200.6	−6.0
6	(1;3′)	193.3	(1;3′)	200.6	−3.8
7	(1;5)	233.7	(1;5)	232.4	0.6
8	(1;5′)	239.1	(1;5′)	232.4	2.8
9	(1;2)	268.9	(2;5)	314.6	−17.0
10	(1;2′)	275.5	(2;5′)	314.6	−14.2
11	(1;6)	313.7	(1;6)	325.8	−3.8
12	(1;6′)	327.8	(1;6′)	325.8	0.6
13	(2;5)	321.6	(1;2)	337.8	−5.0
14	(2;5′)	-	(1;2′)	337.8	
15	(2;4)	353.6	(2;4)	346.0	2.2
16	(2;4′)	356.7	(2;4′)	346.0	3.0

## Data Availability

Not applicable.
